# A combined experimental and theoretical investigation on cellular blebbing

**DOI:** 10.1038/s41598-017-16825-0

**Published:** 2017-11-30

**Authors:** Chao Fang, T. H. Hui, X. Wei, X. Shao, Yuan Lin

**Affiliations:** 1 Department of Mechanical Engineering, The University of Hong Kong, Hong Kong SAR, China; 2 HKU-Shenzhen Institute of Research and Innovation (HKU-SIRI), Shenzhen, Guangdong, China

## Abstract

Although accumulating evidence has demonstrated the important role of membrane blebbing in various cellular processes, the fundamental question of how the initiation/evolution of blebs are influenced by physical factors like membrane-cortex interactions and intracellular pressure remains unclear. Here, we report a combined modeling and experimental study to address this outstanding issue. Specifically, boundary integral method was used to track the motion of membrane (in 3D) during blebbing while possible rupture of the bilayer-cortex adhesion has also been taken into account. We showed that, for a given differential pressure across the cell membrane, the size of the weakened cortex must be over a critical value for blebbing to occur and the steady-state volume of a bleb is proportional to its initial growth rate, all in good agreement with recent experiments. The predicted shape evolution of blebs also matches well with our observations. Finally, a blebbing map, summarizing the essential physics involved, was obtained which exhibits three distinct regimes: no bleb formation corresponding to a low intracellular pressure or a small weakened cortex region; bleb formed with a fixed width when the disrupted cortex zone is very large; and a growing bleb resulted from progressive membrane-cortex detachment under intermediate weakened cortex size.

## Introduction

Cellular blebs are membrane protrusions caused by the detachment of the lipid bilayer from the underlying actin cortex. Besides serving as a hallmark for apoptosis, blebbing is also believed to play a role in various processes like cell spreading^1^ and cytokinesis^2^. For example, it has been suggested that blebs are critical for cells to maintain membrane tension homeostasis^3^. Recent evidence also indicated that migrating cells might have utilized blebbing in regulating their movement^4–9^. For these reasons, intense research effort has been invested to understand how cell blebbing takes place as well as identify key factors governing this process. Specifically, it is well known now that the outgrowth of cell membrane (on regions with a weakened cortex) is driven by the intracellular hydrostatic pressure. The initially formed bleb can continue to grow, aided by the successive disruption of membrane-cortex adhesion, until a steady state size is reached^4,10^. Interestingly, within a few minutes, the cortex can be re-formed underneath the bulged membrane which restores the actin contractility locally and eventually leads to the total retraction of the bleb^11^. Theoretically, several attempts have also been made to describe the formation and evolution of cellular blebs. For instance, by assuming that the cross-membrane pressure difference is balanced by the passive tension in the lipid bilayer and the active cortical contraction collectively, a simple model was developed to predict the size of blebs as well as the threshold cortical tension for blebbing to take place^12^. In addition, the formation of blebs has also been analyzed by treating the membrane as an elastic shell^13–17^. Recently, following a different approach, the shape evolution of a cellular bleb was described by tracking the flow of cytosol^18–21^ as well as the transport and assembly of actins^22^.

It must be pointed out that many simplifications were made in these aforementioned investigations. For example, many studies focused on the static scenario only^12,23^ and the bleb is basically assumed to be spherical^12,13,17,23^. In comparison, although the blebbing dynamics was considered in several models by tracking the flow of cytosol^18–22^, the problem was examined in the two-dimensional configuration. Furthermore, despite that membrane-cortex connection was taken into account in previous studies on predicting the bleb shape^14–16,20,21^ or blebbing-assisted migration of cells^19^, the regulation of such interaction on the dynamics of blebbing (like how fast the bleb can grow and the critical pressure for blebbing to occur) has not been thoroughly examined. In reality, an initially formed bleb can continue to increase in size via successive rupture of bilayer-cortex adhesion and assume a non-spherical shape because of transverse shear in the membrane and its cohesion with the cortex. Unfortunately, a theoretical 3D model capable of capturing these important features is still lacking. In addition, fundamental questions like how the formation and evolution of blebs are governed by key physical factors such as dynamic intracellular pressure level and initial cortex weaken size all remain unclear.

Here we report a combined modeling and experimental study to address these issues. Specifically, boundary integral method was used to track the motion of membrane (in 3D) during the blebbing process while possible rupture of the bilayer-cortex adhesion has also been considered. First of all, the predicted shape evolution of blebs from our model matches well with our experimental observations. Interestingly, it was also found that, for a given weakened size of the cortex, a threshold intracellular pressure is needed for bleb formation and the steady-state volume of a bleb is linearly proportional to its initial growth rate, all in well agreement with recent experiments^12,24^. Finally, a blebbing map, summarizing the essential physics involved, was obtained which exhibits three distinct regimes: no bleb formation (corresponding to a very low intracellular pressure or a small weakened cortex region); bleb formed with a fixed width when the disrupted cortex is large, and a growing bleb resulted from progressive membrane-cortex detachment under intermediate weakened cortex size.

## Theoretical Model for Cellular Blebbing

**Figure 1:**
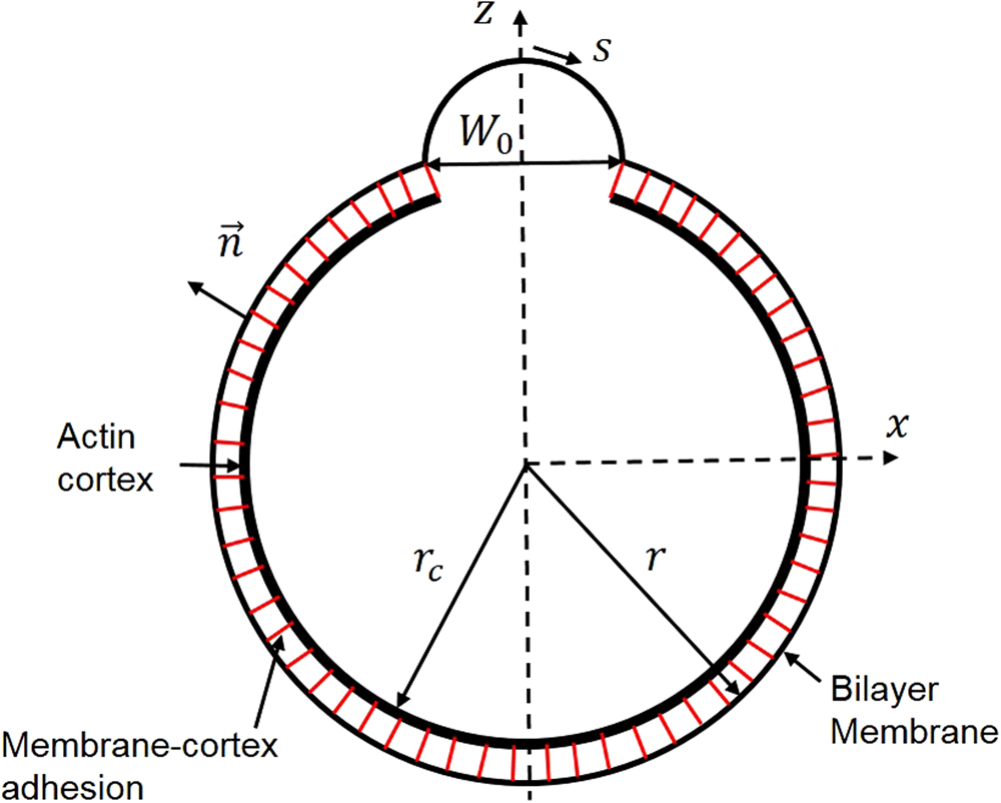
Schematic plot of a cellular bleb. The actin cortex is treated as rigid and stationary because of its high bending rigidity. *r*_*c*_ and *r* are the initial radii of the actin cortex and bilayer membrane, respectively. *W*_0_ represents the size of the region where cortex is disrupted.

Consider the formation a membrane bleb over a region (with width *W*_0_) where the cortex of a spherical suspension cell is disrupted, refer to Fig. 1. For simplicity, we proceed by assuming that the cytosol and culture fluid have the same viscosity (*μ*). As such, their movements can all be described by^25^:

1a $$\mu \frac{{\partial }^{2}{u}_{i}(\overrightarrow{y})}{\partial {y}_{j}^{2}}-\frac{\partial p(\overrightarrow{y})}{\partial {y}_{i}}+\overrightarrow{f}(\overrightarrow{y})=0$$

1b $$\frac{\partial {u}_{i}(\overrightarrow{y})}{\partial {y}_{i}}=0$$

where *μ* represents the viscosity of cytosol (and the external fluid), $\overrightarrow{y}$ refers to the position vector, *u*_*i*_, *p* and $\overrightarrow{f}$ are the fluid velocity along the *i*-th direction, pressure and the force density (arising from membrane elasticity and membrane-cortex adhesion) acting on the fluid by the membrane respectively. Note that the inertia effect has been neglected here given the over-damped nature of the system (i.e. the Stokes flow assumption). In addition, the flow field induced by blebbing is expected to vanish when moving far away from the cell, that is:

2 $${u}_{i}=0,\,|\overrightarrow{y}|\to \infty $$

The cell membrane separating the cytosol and the medium can be treated as an immersed boundary, imposing the following conditions to the flow fields

3a $${[\overrightarrow{u}]}_{s}=0$$

3b $${[{\sigma }_{ij}{n}_{i}]}_{s}={n}_{j}{\rm{\Delta }}\sigma $$

where []_*s*_ stands for the jump of the function across the membrane (from the outside to the inside of cell), $\overrightarrow{n}$ is the out-normal of the membrane surface *S*, *σ*_*ij*_ is the stress tensor within the fluid taking the form ${\sigma }_{ij}=-p{\delta }_{ij}+(\frac{\partial {u}_{i}}{\partial {y}_{j}}+\frac{\partial {u}_{j}}{\partial {y}_{i}})$. Physically, equation (3a) means that the velocity field of the fluid must be continuous across the membrane, as well as satisfy the no-slip boundary condition there, while equation (3b) comes from the force equilibrium of the membrane. From the boundary integral approach, the velocity field can be expressed as^25^

4 $${u}_{i}(\overrightarrow{\xi })={\iint }_{S}{u}_{i}^{j}(\overrightarrow{\xi },\overrightarrow{y}){n}_{j}(\overrightarrow{y}){\rm{\Delta }}\sigma (\overrightarrow{y})d{S}_{\overrightarrow{y}}$$

where

5 $${u}_{i}^{j}(\overrightarrow{\xi },\overrightarrow{y})=-\frac{1}{8\pi \mu }(\frac{{\delta }_{ij}}{r}+\frac{({\xi }_{i}-{y}_{i})({\xi }_{j}-{y}_{j})}{{r}^{3}}),\,r=|\overrightarrow{\xi }-\overrightarrow{y}|$$

is the so-called fundamental solution of the Stokes equation, located at point $\overrightarrow{y}$ and oriented in the *j*-th direction. The term Δ*σ* appeared in equation (4) contains contributions from the bilayer membrane stretching *σ*_*m*_, transverse membrane shear *σ*_*s*_ and membrane-cortex adhesion *σ*_*a*_, i.e.

6 $${\rm{\Delta }}\sigma ={\sigma }_{m}+{\sigma }_{s}+{\sigma }_{a}$$

In this setup, all the components of Δ*σ* (i.e. *σ*_*m*_, *σ*_*s*_ and *σ*_*a*_) are assumed to be along the normal direction of the membrane. Furthermore, if the bilayer membrane is treated as linear elastic, then the tension γ_*m*_ inside can be related to the membrane area $A$ as^26–28^

7 $${\gamma }_{m}={K}_{A}\frac{A-{A}_{0}}{{A}_{0}}$$

where *K*_*A*_ is the so-called area expansion modulus and *A*_0_ is unstressed membrane area. Notice that, here the lipid membrane is basically assumed to have a uniform (equal-biaxial) tension everywhere, a treatment that is consistent with recent evidence that lipid flow takes place within milliseconds^29^ and, hence, any blebbing-induced local stretch can quickly be distributed to the entire membrane. From Young-Laplace law, we have

8 $${\sigma }_{m}={\gamma }_{m}({C}_{1}+{C}_{2})$$

where *C*_1_ and *C*_2_ stands for two principle curvatures of the curved membrane that can be expressed as ${C}_{1}=-\frac{x^{\prime} z^{\prime\prime} -z^{\prime} x^{\prime\prime} }{{(x{^{\prime} }^{2}+z{^{\prime} }^{2})}^{3/2}}$ and ${C}_{2}=-\frac{z^{\prime} }{x}$ ^30^. Here, *x*′, *z*′, *x*″, *z*″ are derivatives of s, where *s* is the arclength coordinate along the membrane (with *s* = 0 corresponding to the zenith point of the cell), *x*(*s*) is the distance from the symmetric axis at point *s* while *z*(*s*) represents the vertical distance relative to the center of cortex (Fig. 1).

From the shell theory, σ_*s*_ can be calculated as^31^

9 $${\sigma }_{s}=-\frac{1}{x}\frac{\partial (xq)}{\partial s}$$

where

10 $$q=\frac{1}{x}\frac{\partial x}{\partial s}[\frac{\partial (x{m}_{1})}{\partial x}-{m}_{2}]$$

is the transverse shear within membrane, ${m}_{1}={K}_{b}{C}_{1}$ and ${m}_{2}={K}_{b}{C}_{2}$ with *K*_*b*_ being the bending rigidity of membrane.

A linear cohesive law was used in the present study to describe membrane-cortex adhesion, that is *σ*_*a*_ is assumed to take the form

11 $${\sigma }_{a}=\{\begin{array}{cc}{\sigma }_{c}\cdot \frac{\delta }{{\delta }_{c}}, & 0 < \delta  < {\delta }_{c}\\ 0, & \delta \ge {\delta }_{c}\end{array}$$

where *σ*_*c*_ is the maximum traction (along the normal direction) the membrane-cortex adhesion can sustain while *δ*_*c*_ and *δ* are the critical and real separation between the bilayer membrane and actin cortex, respectively. Evidently, the quantity $\frac{1}{2}{\sigma }_{c}{\delta }_{c}$ represents the bilayer-cortex adhesion energy density which has been estimated to be of the order of ${10}^{-6} \sim {10}^{-5}J/{m}^{2}$ ^32^. Given that the cortex should be much stiffer than the lipid bilayer, its deformation is neglected here for simplicity (i.e. the cortex is assumed to be rigid).

With these descriptions at hand, the membrane velocities at time *t* + Δ*t* can be calculated from equation (4) based on its velocity field at the previous time step *t*. Specifically, to address the singularity issue in equation (5) (as $\overrightarrow{y}$ approaches $\overrightarrow{\xi }$), we rewrite equation (4) as^33^

12 $${u}_{i}(\overrightarrow{\xi })={\iint }_{S}{u}_{i}^{j}(\overrightarrow{\xi },\overrightarrow{y}){n}_{j}(\overrightarrow{y})[{\rm{\Delta }}\sigma (\overrightarrow{y})-{\rm{\Delta }}\sigma (\overrightarrow{\xi })]d{S}_{\overrightarrow{y}}+{\rm{\Delta }}\sigma (\overrightarrow{\xi }){\iint }_{S}{u}_{i}^{j}(\overrightarrow{\xi },\overrightarrow{y}){n}_{j}(\overrightarrow{y})d{S}_{\overrightarrow{y}}$$

Note that, the first term on the right hand side of equation (12) is a regular integral now while the second term vanishes for any closed shape of membrane (see^33^ for details). Therefore, we have

13 $${u}_{i}(\overrightarrow{\xi })={\iint }_{S}{u}_{i}^{j}(\overrightarrow{\xi },\overrightarrow{y}){n}_{j}(\,\overrightarrow{y})[{\rm{\Delta }}\sigma (\,\overrightarrow{y})-{\rm{\Delta }}\sigma (\overrightarrow{\xi })]d{S}_{\overrightarrow{y}}$$

## Results

Based on our experimental observations, the width *W*_0_ of the initially weakened cortex region was chosen to be 2.63 *um*. The values of other parameters (unless stated otherwise) are listed in Table 1.

**Table 1:** Parameters list of boundary integral model.

Symbol	Physical meaning	Value	Ref
*r*	Initial radius of bilayer membrane	10 *um*	Our experiment
*r*_*c*_	Radius of actin cortex	9.9 *um*	Estimated
*W*_0_	Width of the initially weakened region	2.63 *um*	Our experiment
*μ*	Cytosol dynamic viscosity	8 Pa · s	43
Δ*σ*	Differential pressure	33.6 Pa	Our experiment
*γ*	Bilayer membrane tension	5 *pN/um*	44,45
*K*_*A*_	Areal expansion modulus	40 *pN/um*	42
*K*_*b*_	Bending rigidity	10~20 *k*_*b*_ *T*	35
*σc*	Maximum adhesion traction	125 Pa	32
*δ*_*c*_	Critical membrane-cortex separation	0.04 *um*	46

**Figure 2:**
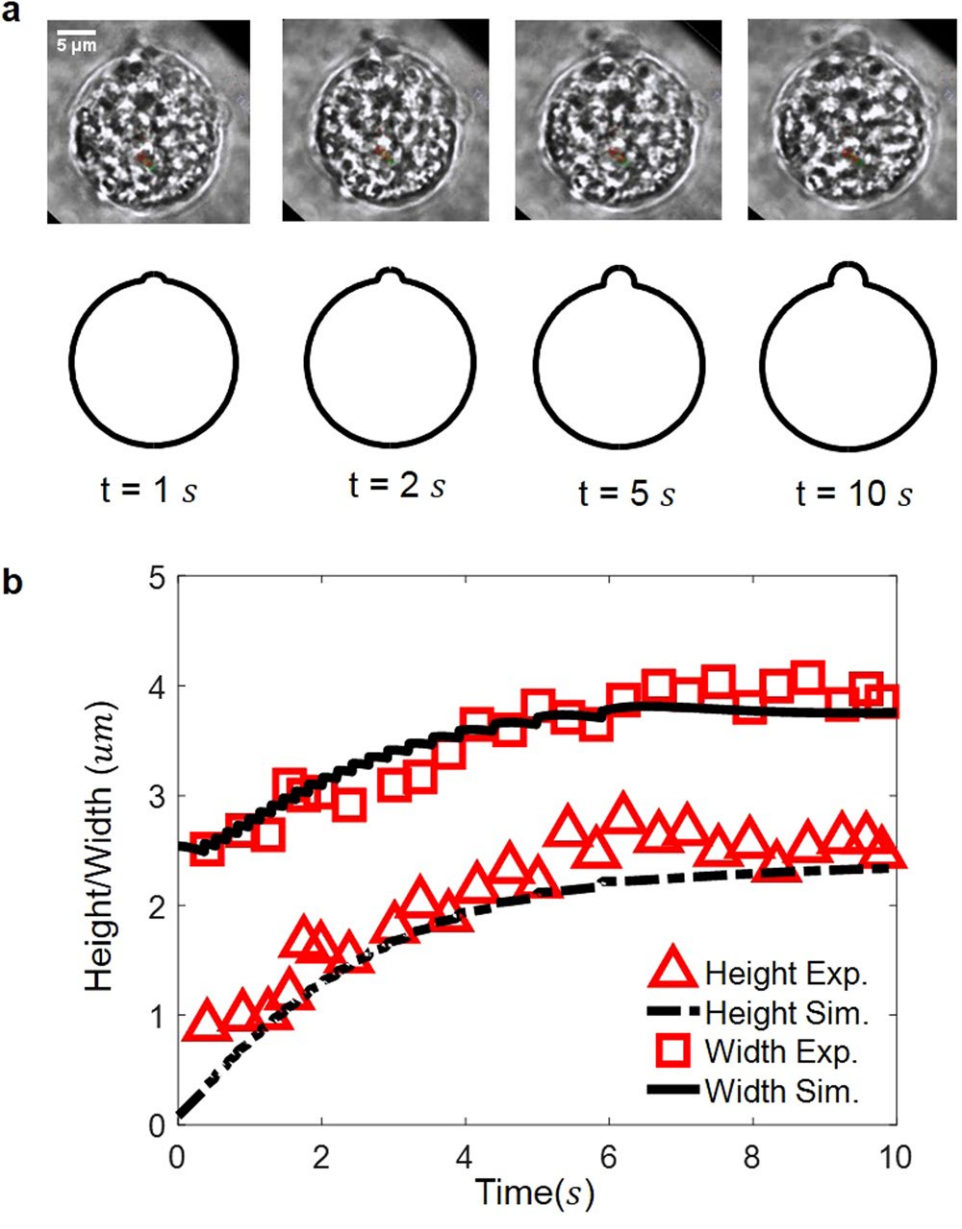
**(a)** Comparison between the observed bleb profile (top pannel), captured by phase contrast microscopy in our experiment, and the predicted bleb shapes (bottom pannel). **(b)** Evolution of the bleb height and width where simulation and experimental results are represented by lines and symbols, respectively. Sudden jumps in the simulation curve come from the failure of individual cohesive element.

Our simulation showed that the bilayer membrane bulges first on the weakened part and then forms a bleb whose size continues to grow (aided by progressive membrane-cortex detachment) before a steady state is reached, refer to Fig. 2. In comparison, the shape and size evolution of blebs observed in our experiment are also shown in Fig. 2. Clearly, we can see that good agreement between simulation and experiment has been achieved.

**Figure 3:**
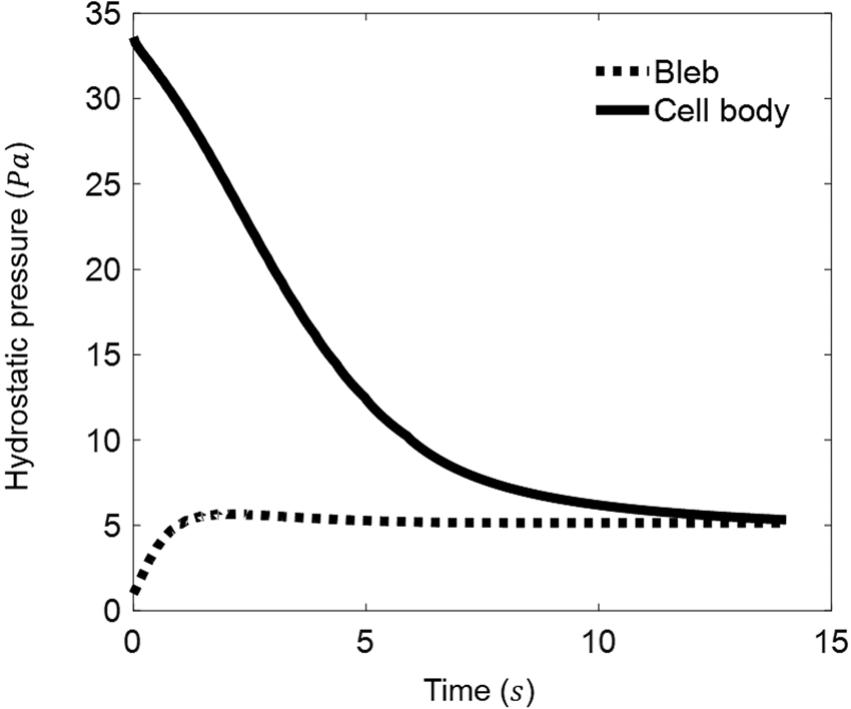
Evolution of the cell body pressure (Δ*σ*) and the bleb pressure (defined as *σ*_*m*_ + *σ*_*s*_ in equation (6)) during the blebbing process.

It is well known that the growth of bleb is driven by the unbalanced differential pressure (Δ*σ*), between inside and outside of the cell, pushing the cytosol into the bleb. Initially, such unbalanced pressure is maximum because of the sudden removal of the cortex (and hence the membrane-cortex adhesion *σ*_*a*_, see equation (6)). As the bleb grows, the intracellular pressure gradient drops due to the viscous flow of the cytosol. At the same time, the principal curvatures (*C*_1_ and *C*_2_) of the blebbed region, along with the overall membrane area (*A*), increase leading to elevated membrane forces *σ*_m_ and σ_*s*_ on the right side of equation (6). Eventually, Δ*σ* (referred to as the pressure in the cell body in Fig. 3) will be completely offset by bleb membrane forces ${\sigma }_{m}+{\sigma }_{s}$ when the steady-state is reached (Fig. 3).

### Bleb formation regulated by intracellular pressure and membrane-cortex adhesion

**Figure 4:**
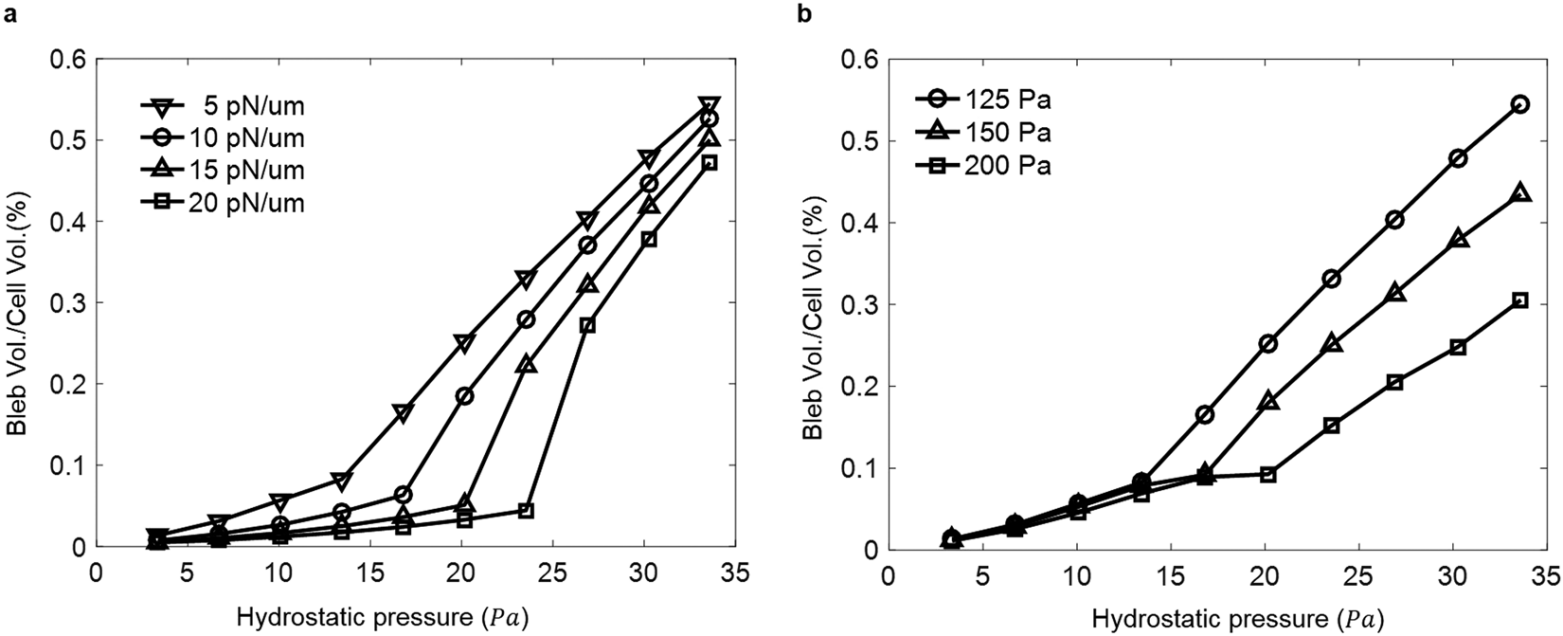
The critical differential pressure for bleb formation is influenced by both the bilayer membrane tension (**a**) and membrane-cortex adhesion strength (**b**). When Δ*σ* is beyond a threshold value, membrane-cortex adhesion begins to fail and propagates, resulting in a significant growth in the steady-state bleb volume (normalized by the initial cell volume).

Previous studies have shown that the magnitude of differential pressure (Δ*σ*) plays a key role in bleb formation^12^. Specifically, for a spherical cell with radius *r*, this pressure will be balanced by the total membrane tension T through the Young-Laplace law as ${\rm{\Delta }}\sigma =2T/r$ with $T={\gamma }_{c}+\gamma$ where *γ* represents the tension in the lipid bi-layer while *γ*_*c*_ stands for cortical tension originated from actomyosin contraction. However, *γ*_*c*_ vanishes in the region where cortex is disrupted, which eventually leads to the outgrowth of the bi-layer. By assuming the bleb possesses a spherical geometry, Tinevez and co-workers estimated the critical value of Δ*σ* for bleb formation to be ${\rm{\Delta }}\sigma  \sim 4\gamma /{W}_{0}$, with *W*_0_ being the size of the weakened region of cortex^12^. That is, no apparent bleb will be formed if the differential pressure is below this threshold level. Interestingly, similar trend was also revealed by our simulations. In particular, as shown in Fig. 4a, the steady-state bleb volume became significant only when the intracellular pressure is above a critical level. Furthermore, not surprisingly, the value of this threshold pressure increases with the bi-layer tension, i.e. when the percentage of cortical contraction in the overall membrane tension decreases. Specifically, when $\gamma =15pN/um$ and ${W}_{0}=2.63\,um$, the critical Δ*σ* was found to be around 22.08 *Pa* from our simulations [Fig. 4a], in good agreement with the value (~20 *Pa*) estimated by Tinevez *et al*.^12^. The small difference may come from fact that possible failure of membrane-cortex adhesion (i.e. the breaking of membrane-cortex “links” as schematically shown in Fig. 1) was taken into account in our formulation. That is, *W*_0_ can grow in our simulations while this quantity was fixed in the simple estimation model^12^. In this regard, it is conceivable that the critical Δ*σ* for bleb formation is also regulated by the strength (*σ*_*c*_) of membrane-cortex cohesion. Indeed, as illustrated in Fig. 4b, a higher pressure level must be reached to trigger blebbing as *σ*_*c*_ increases.

**Figure 5:**
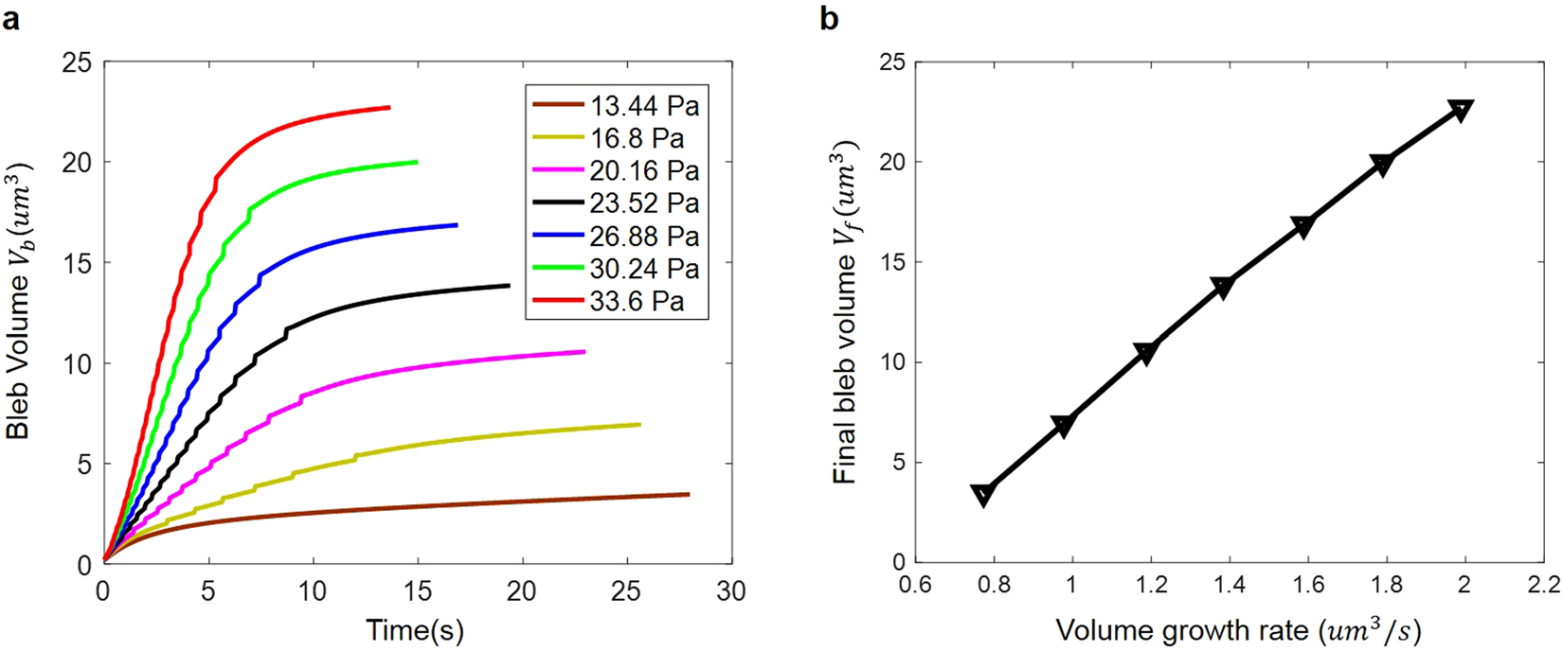
**(a)** The bleb volume (*V*_*b*_) as a function of time under different differential pressure ${\rm{\Delta }}\sigma$. **(b)** Steady-state bleb volume (*V*_*f*_) is proportional to its initial growth rate, i.e. ${V}_{f} \sim d{V}_{b}/dt$ as $t\to 0$.

Another interesting observation from previous investigations is that the final bleb volume seems to be proportional to its initial growth rate^24^. To see whether similar conclusion can be obtained from our model, we tracked the volume evolution of blebs formed under different intracellular pressure. Obviously, the bleb grows faster as Δ*σ* increases as shown in Fig. 5a. By plotting the volume growth rate within the first 0.2 s against the steady-state bleb volume, a linear relationship was indeed observed [Fig. 5b].

**Figure 6:**
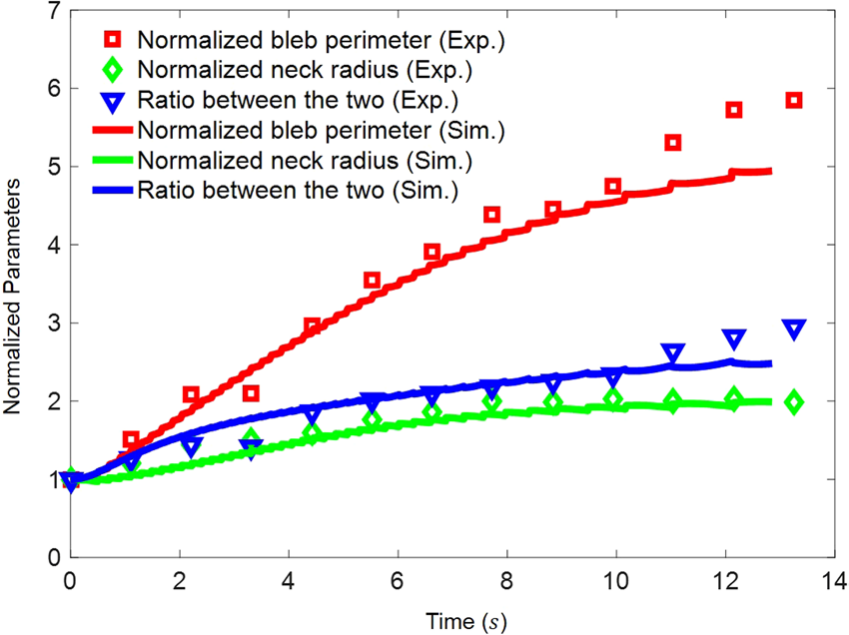
Evolution of the bleb perimeter (i.e. arc-length in 2D) and neck radius. Experiment data shown here are extracted from the work by Charras *et al*.^32^.

To further test the model, we have compared our simulations to another set of experiments^32^ where the perimeter and neck radius of a growing bleb in M2 cell as functions of time were carefully measured (refer to the square and diamond symbols in Fig. 6). In comparison, these quantities predicted from our model are also shown in Fig. 6 by solid lines. Evidently, excellent agreement between theory and experiment has been achieved. It must be pointed out that the membrane tension, initial weakened size and adhesion energy were chosen as $\gamma =2pN/um$, ${W}_{0}=1.5\,um$, and ${\rm{\Gamma }}=5\times {10}^{-6}J/{m}^{2}$ in this simulation, respectively, which are all well within the range reported/estimated^32^ (i.e $\gamma \sim 0.8\mbox{--}6\,pN/um$, ${W}_{0}\sim 1.3\mbox{--}1.6\,um$, ${\rm{\Gamma }}\sim 1.3\mbox{--}9.8\times {10}^{-6}\,J/{m}^{2}$). In addition, the fact that the observed neck radius (of the bleb) kept increasing indicates that gradual detachment between the lipid membrane and cortex indeed took place during the blebbing process, a feature that has been captured by the present model.

### Blebbing map

**Figure 7:**
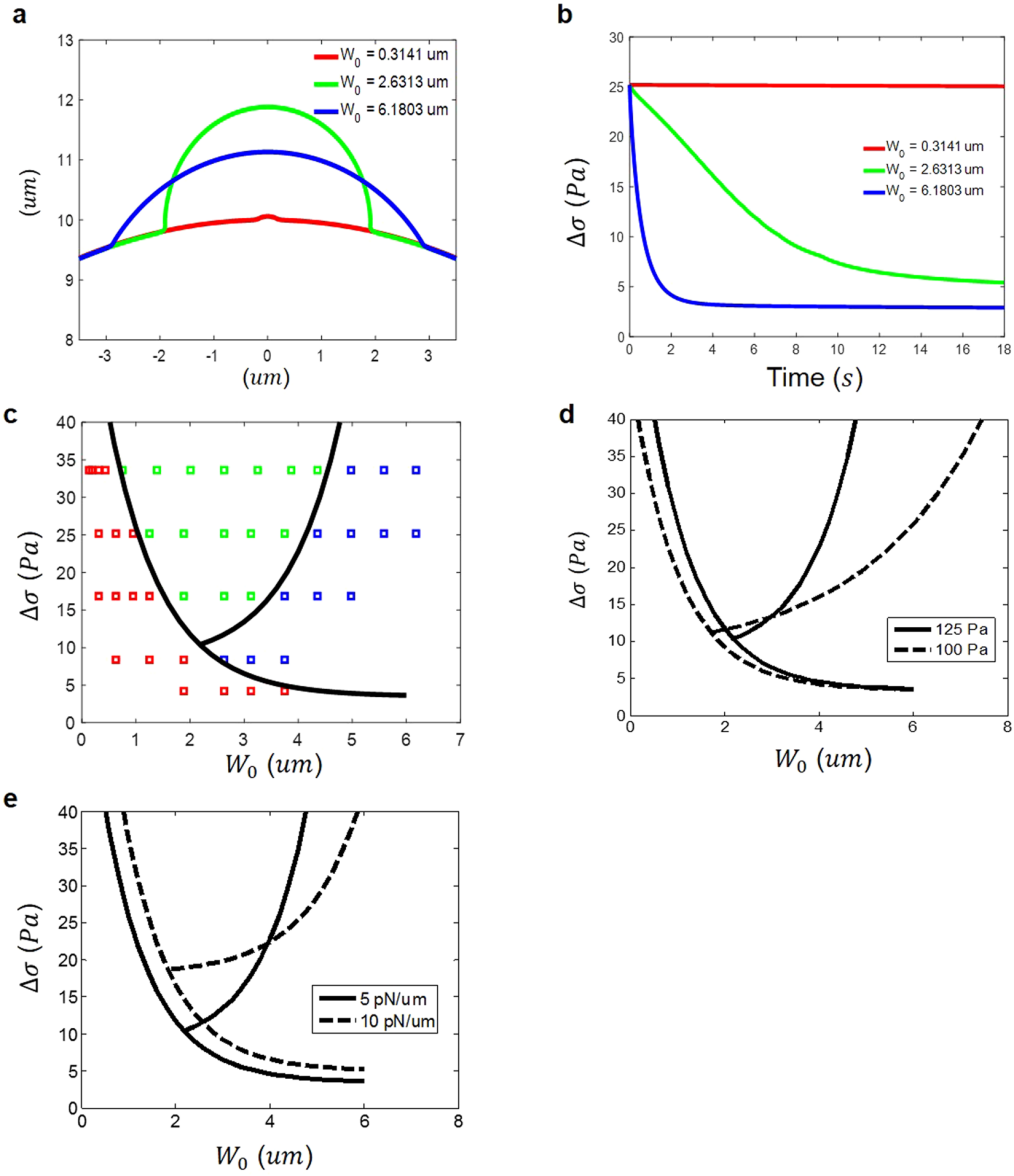
**(a)** Final profile of the bleb under different initial weakened size of cortex *W*_0_. **(b)** Temporal evolution of the differential pressure under different values of *W*_0_. **(c)** Three different scenarios: no apparent blebbing (red), bleb formation with a growing (green) or fixed (blue) width. Varying the membrane-cortex adhesion strength **(d)** or bilayer tension **(e)** will shift the boundaries of these three regions slightly without altering the main features.

Besides differential pressure and cortex-membrane cohesion, it is natural to believe that the initial size of the weakened cortex also plays a key role in the formation of blebs. For example, by considering the pressure-driven peeling of membrane from the cortex, Alert and Casademunt found that a bleb can only be nucleated from a weakened region with size over 100–200 nm^34^. However, the influence of cytosol flow was neglected there. Here, a parametric study on the blebbing process is conducted to unambiguously address this issue. Specifically, the steady-state shapes of the deformed membrane under different weakened size *W*_0_ are shown in Fig. 7a.

Clearly, when *W*_0_ is small, the bilayer membrane can only bulge slightly, that is without apparent bleb formation [Fig. 7a]. In comparison, as the weakened size increases a bleb will form and actually keep growing (by inducing successive membrane-cortex separation) before reaching a steady state size. Interestingly, when *W*_0_ becomes relatively large no rupture of membrane-cortex adhesion will be triggered and the bleb will be developed with a fixed width. To understand these intriguing findings, we examined the temporal evolution of the differential pressure ${\rm{\Delta }}\sigma (t)$ during the blebbing process. As shown in Fig. 7b, negligible change in this quantity took place under small *W*_0_, reflecting the fact that very little membrane bulging and hence fluid movement were resulted in this case. On the other hand, under intermediate weakened size, a gradual decrease in Δ*σ* was observed indicating significant cytosol flow has been triggered to sustain the growth of the bleb. However, a sharp drop in the differential pressure occurred when *W*_0_ is large, presumably because a huge volume of cytosol has flowed into the bleb. Such big drop in Δ*σ* was likely to be the reason for no additional rupture of membrane-cortex adhesion during the blebbing process. These results are best summarized in the so-called blebbing map as shown in Fig. 7c. Essentially, when the initial differential pressure is low, the system will transit from no apparent blebbing to the formation of bleb with a fixed width as *W*_0_ increases. However, under relatively large initial value of Δ*σ*, the scenario of a bleb formed with an increasing width appears at intermediate *W*_0_. Changing the strength of membrane-cortex adhesion or the bi-layer tension level will only shift the boundaries of these three regions slightly [Fig. 7d and e], but maintain these main features mentioned above.

### Effect of membrane bending rigidity

**Figure 8:**
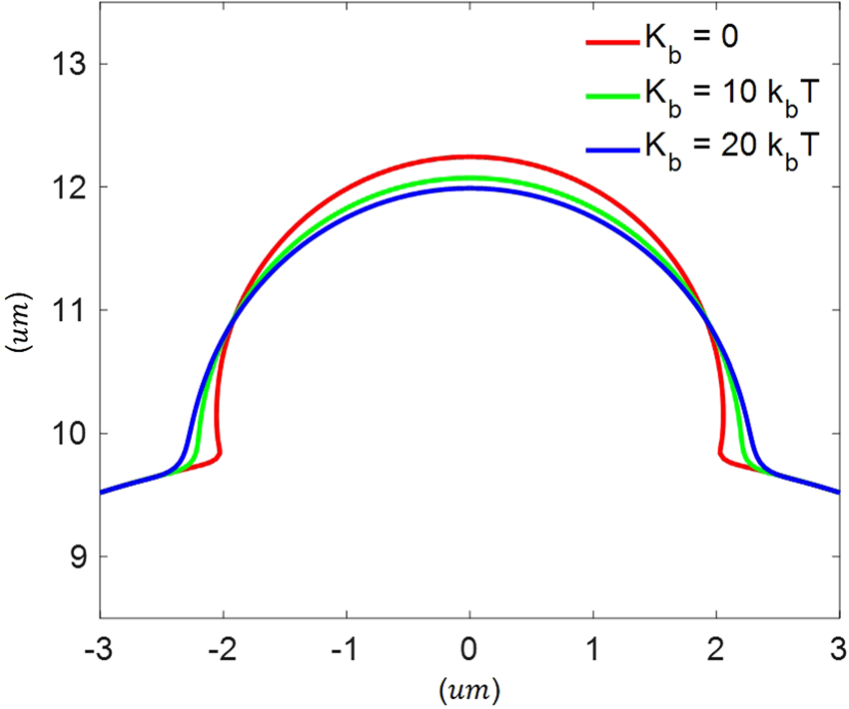
Steady-state shape of blebs under different membrane bending rigidities.

The present model also allows us to examine how cellular blebbing is influenced by the bending rigidity of the bilayer membrane, although its value is commonly believed to be in the range of 10–20 *k*_*B*_ *T* ^35^ with *k*_*B*_ *T* being the thermal energy. As illustrated in Fig. 8, a sharp angle between the bilayer and the un-blebbed part of membrane (i.e. with cortex support underneath) was formed at the edge of the bleb when the bending of the bilayer is neglected. However, a much smoother transition was observed once a non-vanishing bending rigidity is used. Energetically, this is not surprising because a sharp angle corresponds to large membrane curvatures locally, eventually leading to an exceedingly high bending energy stored inside. These results also indicate that factors like bilayer tension and membrane-cortex adhesion should play a more significant role, compared to membrane bending, in determining the steady-state bleb size.

## Discussion

In conclusion, we have presented a combined modeling and experimental study on the phenomenon of cell blebbing here. By taking into account possible rupture of the bilayer-cortex adhesion and the 3D nature of a cellular bleb, the roles of different physical factors such as intracellular pressure, the size of the weakened cortex and bending rigidity of bilayer membrane in the blebbing process have all been elucidated. Specifically, we presented that, for a given weakened size of the cortex, a threshold differential pressure across the membrane is needed for bleb formation and the steady-state volume of a bleb is linearly proportional to its initial growth rate, all in well agreement with experiments^12,24^. Moreover, a blebbing map, summarizing the essential physics involved, was obtained which shows that there three distinct scenarios: no bleb formation (corresponding to a very low intracellular pressure or a small weakened cortex region); bleb formed with a fixed width when the disrupted cortex is large, and a growing bleb resulted from progressive membrane-cortex detachment under intermediate weakened cortex size. Varying the strength of membrane-cortex adhesion and/or bilayer tension will shift the boundaries of these three regions, making it easier (or harder) for a bleb to be nucleated/formed which again is in broad agreement with recent experimental observations^32^. It must be pointed out that a regime of uncontrollable bleb growth was predicted in some previous studies^12,21^ when the membrane stiffness is relative low (or, equivalently, when the intracellular pressure is very high). However, no such phenomenon was observed in our simulations even when *K*_*A*_ was varied from 40 to 1 pN/µm. We believe the main reason is that, unlike in ref.^21^, gradual detachment between the cortex and bilayer membrane was allowed in our model, which effectively serves a dissipation mechanism to absorb the unbalanced intracellular pressure during the blebbing process. As a result, the cell body pressure dropped rapidly in our simulations (refer to Fig. 3), eventually leading to blebs with a steady-state size. Interestingly, it seems that similar finding has also been obtained in the study by Lim and co-workers^19^, where (like the present study) cortex-membrane adhesion/rupture was taken into account and no uncontrollable bleb growth was reported.

Given the important role of membrane blebbing in various cellular processes, this work may help us understand how cells execute their biological duties. For example, evidences have suggested that processes like turnover of cell-extracellular matrix adhesions, cortex remodeling and blebbing are tightly coordinated during the locomotion of cells^6–9^. In addition, it is also believed that, as part of the membrane recycling system, cell blebbing works with endocytosis and exocytosis for cells to achieve homeostasis (in terms of membrane tension or the intracellular pressure). Interestingly, cortex rearrangement often takes place over minutes^36,37^ while the frequency of exocytosis in a non-migrating cell is usually less than one per minute^38^, indicating that these factors may not be very important in the rapid blebbing process (taking ~10–20 seconds to complete). However, they can certainly play a role in related phenomena like the nucleation/retraction of blebs^39^, cell motility and homeostasis regulation of cells. It is conceivable that our model can serve as a platform to systematically examine these issues. Finally, the blebbing map obtained here could be useful in the design of (and be tested by) future experiments. In particular, it will be interesting to see, by varying quantities like membrane tension and the strength of cortex-membrane adhesion experimentally, whether the occurrence and transition of different blebbing modes predicted here can indeed be observed. Such information will undoubtedly enhance our understanding of this intriguing phenomenon. Indeed, investigations along these lines are underway.

## Materials and Methods

### Cell culture

K562 cells, derived from a chronic myeloid leukemia patient with blast crisis^40,41^ were cultured in DMEM medium (Gibco) containing 10% fetal bovine serum (Invitrogen). To mimic the native biological environment *in vivo*, the experiment was conducted with the presence of 5% CO_2_ supply (Warner Instruments) and the temperature was controlled by a temperature regulator (Warner Instruments) at 37 degree Celsius.

### Blebbing induction and analysis

To induce cortical-weakened bleb formation^8^, K562 cells were exposed to cytochalasin D which is known to disrupt the actin cytoskeleton and hence promote bleb nucleation^11,32^. Specifically, 5 ${\rm{\mu }}M$ of cytochalasin D was added to the culture medium of K562 cells for 5 minutes. Once a cellular bleb started to nucleate, its shape evolution was closely monitored by an optical microscopy with 1000X magnification (Nikon) from which the bleb height and width were estimated by the software ImageJ.

### Membrane tension/Differential pressure measurement

Micropipette aspiration was used to measure the overall membrane tension (or equivalent the initial differential pressure) of cells^42^. Specifically, the tips of glass micropipettes, prepared by micropipette pullers (Sutter Instrument), were polished with a microforge (Nikon) to achieve a desired inner radius (*R*_*p*_). The micropipette was then mounted on a motorized stage and connected to a syringe providing suction pressures. An alcoholic multi-tube manometer (tecquipment) was used to record the negative pressure produced by the syringe. During the test, the pipette was first loaded at the same depth of the targeted cell and then moved horizontally to approach it. The suction pressure was applied when an initial tip-cell separation of 30 *um* was reached. Once the cell was grabbed by the micropipette, we gradually increased the suction pressure until it reaches a critical value *P*_*c*_, beyond which the whole cell will be sucked into the pipette spontaneously. Under this critical pressure, the portion of the cell being sucked into the pipette will approximately form a hemisphere (with radius *R*_*p*_) while the portion remained outside assumes a radius *R*_*c*_. The overall membrane tension *γ*_0_ (i.e. the summation of cortical and bi-layer tensions) can then be related to *P*_*c*_, *R*_*p*_ and *R*_*c*_ as^42^

14 $${\gamma }_{0}=\frac{{P}_{c}}{2}{(\frac{1}{{R}_{p}}-\frac{1}{{R}_{c}})}^{-1}$$

After that, the initial differential pressure Δ*σ* was calculated from *γ*_0_ and the cell radius via the Laplace law.

### Numerical scheme

To evaluate the membrane movement from the integral equation (13), a numerical scheme was developed using MATLAB. Specifically, the entire (axisymmetric) membrane was meshed along the meridional and equatorial directions with 130 and 60 elements, respectively. The elements are quadratic with 3 × 3 nodes each. After that, the blebbing process is simulated by repeating the following steps

1. Calculate the principal curvatures, as well as the bending moments *m*_1_ and *m*_2_, in each element by assuming quadratic dependence of variables on both *x* and *z*;
2. Compute each components of Δ*σ* from equations (7–11);
3. Average/smooth the values of Δ*σ* calculated from different neighboring elements and then evaluate the membrane velocity from equation (13) by Gaussian quadrature integration (see ref.^31^ for more details);
4. Employ the forward Euler method to track the movement of the bilayer membrane.

It must be pointed out that severe element distortion may occur near the bleb neck. Therefore, constant re-meshing was carried out to eliminate possible errors/instability induced by this. In addition, simulations with different element sizes have also been conducted to make sure that results presented here are mesh-size independent^33^.

## References

[1] Norman LL, Sengupta K, Aranda-Espinoza H (2011). Blebbing dynamics during endothelial cell spreading. Eur. J. Cell Biol..

[2] Tokumitsu T, Maramorosch K (1967). Cytoplasmic protrusions in insect cells during mitosis *in vitro*. J. Cell Biol..

[3] Norman LL, Brugés J, Sengupta K, Sens P, Aranda-Espinoza H (2010). Cell blebbing and membrane area homeostasis in spreading and retracting cells. Biophys. J..

[4] Charras G, Paluch E (2008). Blebs lead the way: how to migrate without lamellipodia. Nat. Rev. Mol. Cell Biol..

[5] Lorentzen A, Bamber J, Sadok A, Elson-Schwab I, Marshall CJ (2011). An ezrin-rich, rigid uropod-like structure directs movement of amoeboid blebbing cells. J. Cell Sci..

[6] Bergert M, Chandradoss SD, Desai RA, Paluch E (2012). Cell mechanics control rapid transitions between blebs and lamellipodia during migration. Proc. Natl. Acad. Sci. USA.

[7] Fackler OT, Grosse R (2008). Cell motility through plasma membrane blebbing. J. Cell Biol..

[8] Paluch EK, Raz E (2013). The role and regulation of blebs in cell migration. Curr. Opin. Cell Biol..

[9] Maugis B (2010). Dynamic instability of the intracellular pressure drives bleb-based motility. J. Cell Sci..

[10] Charras GT (2008). A short history of blebbing. J. Microsc..

[11] Charras GT, Hu CK, Coughlin M, Mitchison TJ (2006). Reassembly of contractile actin cortex in cell blebs. J. Cell Biol..

[12] Tinevez JY (2009). Role of cortical tension in bleb growth. Proc. Natl. Acad. Sci. USA.

[13] Woolley TE (2014). Three mechanical models for blebbing and multi-blebbing. IMA J. Appl. Math..

[14] Woolley TE (2014). Cellular blebs: pressure-driven, axisymmetric, membrane protrusions. Biomech. Model. Mechanobiol..

[15] Woolley TE (2015). Global contraction or local growth, bleb shape depends on more than just cell structure. J. Theor. Biol..

[16] Woolley TE, Gaffney EA, Goriely A (2015). Membrane shrinkage and cortex remodelling are predicted to work in harmony to retract blebs. R. Soc. Open Sci..

[17] Woolley TE, Gaffney EA, Goriely A (2017). Random blebbing motion: A simple model linking cell structural properties to migration characteristics. Phys. Rev. E..

[18] Lim FY, Chiam KH, Mahadevan L (2012). The size, shape, and dynamics of cellular blebs. EPL.

[19] Lim FY, Koon YL, Chiam K-H (2013). A computational model of amoeboid cell migration. Comput. Methods. Biomech. Biomed. Eng..

[20] Strychalski W, Guy RD (2012). A computational model of bleb formation. Math. Med. Biol..

[21] Strychalski W, Guy RD (2016). Intracellular pressure dynamics in blebbing cells. Biophys. J..

[22] Young J, Mitran S (2010). A numerical model of cellular blebbing: a volume-conserving, fluid-structure interaction model of the entire cell. J. Biomech..

[23] Hu, J. Mathematical Modeling and Analysis of *in vitro* Actin Filament Dynamics and Cell Blebbing. Ph.D thesis, University of Minnesota (2009).

[24] Cunningham CC (1995). Actin polymerization and intracellular solvent flow in cell surface blebbing. J. Cell Biol..

[25] Power, H. & Wrobel, L. C. *Boundary Integral Methods in Fluid Mechanics*. (Computational Mechanics Publications, 1995).

[26] Secomb TW (1988). Interaction between bending and tension forces in bilayer membranes. Biophys. J..

[27] Fisher JL, Margulies SS (2007). Modeling the effect of stretch and plasma membrane tension on Na+-K+-ATPase activity in alveolar epithelial cells. Am. J. Physiol. Lung Cell Mol. Physiol..

[28] Diz-Muñoz A, Fletcher DA, Weiner OD (2013). Use the force: membrane tension as an organizer of cell shape and motility. Trends Cell Biol..

[29] Keren K (2008). Mechanism of shape determination in motile cells. Nature.

[30] Yi X, Gao H (2015). Cell membrane wrapping of a spherical thin elastic shell. Soft Matter..

[31] Pozrikidis C (2001). Effect of membrane bending stiffness on the deformation of capsules in simple shear flow. J. Fluid Mech..

[32] Charras GT, Coughlin M, Mitchison TJ, Mahadevan L (2008). Life and times of a cellular bleb. Biophys. J..

[33] Pozrikidis, C. *Boundary Integral and Singularity Methods for Linearized Viscous Flow*. (Cambridge University Press, 1992).

[34] Alert R, Casademunt J (2016). Bleb nucleation through membrane peeling. Phys. Rev. Lett..

[35] Boal, D. *Mechanics of the Cell*. (Cambridge University Press, 2012).

[36] Lappalainen P, Drubin DG (1997). Cofilin promotes rapid actin filament turnover *in vivo*. Nature.

[37] Mallavarapu A, Mitchison T (1999). Regulated actin cytoskeleton assembly at filopodium tips controls their extension and retraction. J. Cell Biol..

[38] Gauthier NC, Fardin MA, Roca-Cusachs P, Sheetz MP (2011). Temporary increase in plasma membrane tension coordinates the activation of exocytosis and contraction during cell spreading. Proc. Natl. Acad. Sci. USA.

[39] Collier S, Paschke P, Kay RR, Bretschneider T (2017). Image based modeling of bleb site selection. Sci. Rep..

[40] Koeffler H, Golde D (1980). Human myeloid leukemia cell lines: a review. Blood..

[41] Sawyers CL (1999). Chronic myeloid leukemia. N. Engl. J. Med..

[42] Hochmuth RM (2000). Micropipette aspiration of living cells. J. Biomech. Eng..

[43] Wirtz D (2009). Particle-tracking microrheology of living cells: principles and applications. Annu. Rev. Biophys..

[44] Dai J, Sheetz MP (1999). Membrane tether formation from blebbing cells. Biophys. J..

[45] Simson R (1998). Membrane bending modulus and adhesion energy of wild-type and mutant cells of Dictyostelium lacking talin or cortexillins. Biophys. J..

[46] Liu D (2007). Single-molecule detection of phosphorylation-induced plasticity changes during ezrin activation. FEBS Lett..

